# Gel immersion technique in interventional endoscopic ultrasound for pancreatobiliary lesions

**DOI:** 10.1055/a-2357-2325

**Published:** 2024-07-30

**Authors:** Haruka Toyonaga, Tsuyoshi Hayashi, Kazuki Hama, Toshifumi Kin, Masayo Motoya, Kuniyuki Takahashi, Akio Katanuma

**Affiliations:** 1 37009 Center for Gastroenterology, Teine Keijinkai Hospital, Sapporo, Japan; 2 37009 Gastroenterology and Hepatology, Teine Keijinkai Hospital, Sapporo, Japan

Interventional endoscopic ultrasound (iEUS), including EUS-guided tissue acquisition (EUS-TA) and biliary drainage (EUS-BD), plays a crucial role in the assessment and treatment of pancreaticobiliary lesions. Yet, iEUS presents challenges due to anatomical factors, posing a risk of serious complications. For observational pancreaticobiliary EUS, studies have shown that utilizing a high viscosity gel (Viscoclear; Otsuka Pharmaceutical, Tokushima, Japan) instead of water to fill the duodenal lumen yields clearer EUS images and effectively maintains the luminal dilatation for an extended period 1 2 3 . Recently, a handful of reports have explored the gel immersion technique within iEUS 4 5 . Here, we evaluate the effectiveness and safety of the gel immersion method, particularly in EUS-TA and EUS-BD ( Video 1 ).

The gel immersion technique in interventional endoscopic ultrasonography (iEUS) for pancreaticobiliary lesions.Video 1

EUS-TA was carried out in instances of nonexposed-type ampullary tumors presenting with jaundice, where multiple endoscopic biopsies failed to confirm malignancy. Gel immersion facilitated clear imaging without the need to compress the papilla with the EUS tip or embedding it in the mucosal folds. This approach enabled successful tissue sampling and the detection of malignancy ( Fig. 1 ).

**Fig. 1:**
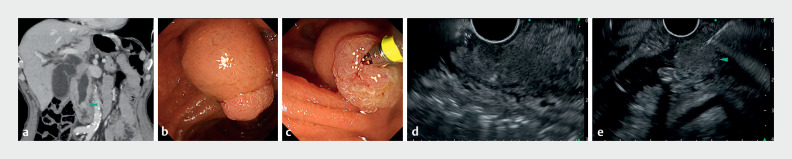
Gel immersion technique in endoscopic ultrasound-guided tissue acquisition (EUS-TA) for ampullary tumors. **a** Computed tomography (CT) showed an ampullary tumor (arrowhead) causing biliary obstruction and jaundice. **b** Endoscopic view of a nonexposed-type ampullary tumor. **c** Despite multiple biopsies, malignancy could not be detected. **d** Efforts were made to visualize the tumor by means of EUS-TA, but despite water injection, the duodenal lumen did not extend, and it was difficult to identify the tumor as it was embedded within the duodenal mucosa. **e** By filling the duodenal lumen with a high viscosity gel and displacing the air, good ultrasound propagation was achieved. The presence of the gel allowed the EUS probe to be distanced from the mucosa, and by extending the mucosal folds, the tumor (arrowhead) was clearly visualized without being compressed, enabling accurate performance of EUS-TA.

When conducting iEUS from the duodenal bulb with a convex-type EUS scope, there can be visibility issues for both the needle and the gastrointestinal wall due to air trapped within the duodenal bulb space. This increases the risk of a “double puncture” involving the gastropyloric ring and the duodenal wall, attributed to the gap between the tip of the EUS probe and the instrument channel. Gel immersion mitigates these risks by displacing the air disrupting ultrasound transmission and extending the duodenal wall for longer periods. Gel immersion is also effective during EUS-TA for pancreatic head cancer via the duodenal bulb ( Fig. 2 ), EUS-guided gallbladder drainage (EUS-GBD) ( Fig. 3 ), and the EUS-guided rendezvous technique for challenging transpapillary biliary cannulation ( Fig. 4 ). This enhances both the visualization and the safety of punctures.

**Fig. 2:**
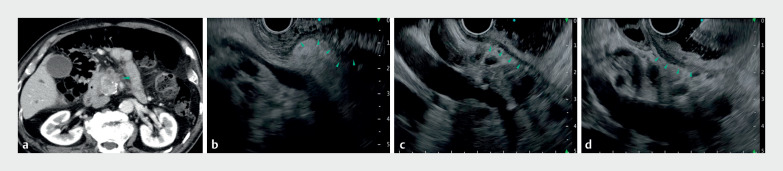
Gel immersion technique in endoscopic ultrasound-guided tissue acquisition (EUS-TA) via the duodenal bulb for a pancreatic head tumor. **a** A hypovascular tumor suspicious for pancreatic head cancer (arrowhead) was identified on contrast-enhanced CT, and EUS-TA was performed. **b** An attempt to perform EUS-TA from the duodenal bulb was hindered by air retention, which obscured needle visibility. Additionally, the tortuosity and bending of the duodenum lumen with concentrated mucosal folds (arrowheads), increased the risk of a double puncture when attempting needle insertion. **c, d** Filling the duodenal bulb with gel allowed the duodenal wall and mucosal folds to extend well and easily. The area where the needle emerged was also clearly visible, eliminating the risk of a double puncture.

**Fig. 3:**
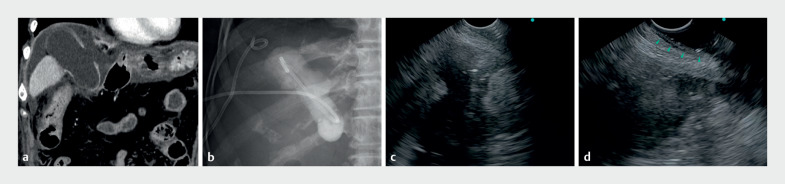
Gel immersion technique in endoscopic ultrasound-guided gallbladder drainage (EUS-GBD). **a** Contrast-enhanced CT revealed severe perforated cholecystitis with disruption of the gallbladder wall. **b** The patient was considered too high risk for a surgical intervention, and so a nonsurgical treatment approach was adopted. Initially, percutaneous drainage was performed, and once the cholecystitis was controlled, internal drainage was established using EUS-GBD. **c** An attempt was made to perform EUS-GBD using a forward-viewing convex EUS scope, but air, easily accumulating in the duodenal bulb, hindered the ultrasound imaging and prevented a clear delineation of the puncture line. **d** With the gel immersion technique, the puncture line was clearly delineated, the duodenal wall was sufficiently extended (arrowheads), and EUS-GBD was safely completed.

**Fig. 4:**
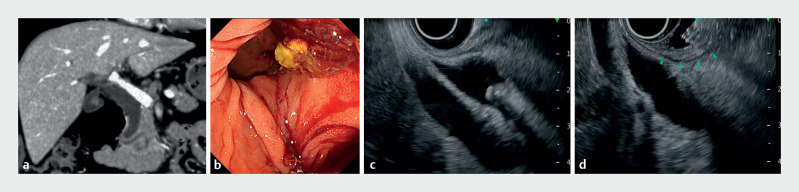
Gel immersion technique with the endoscopic ultrasound-guided rendezvous (EUS-RV) method for a difficult biliary cannulation case. **a** In a patient with cholangitis due to common bile duct stones, contrast-enhanced CT revealed the presence of a large duodenal diverticulum at the papilla. **b** Transpapillary biliary cannulation was attempted, but the duodenal papilla was located within the diverticulum and could not be seen using traction, making cannulation impossible. **c** Although the common bile duct was visualized from the duodenal bulb using EUS, the accumulation of air in the lumen and the distance between the EUS probe tip and the channel prevented EUS imaging of the puncture line. **d** With the gel immersion technique, the duodenal bulb was filled with the gel, and the duodenal mucosa (arrowheads) was sufficiently extended, allowing the safe and successful completion of EUS-RV with clear EUS imaging.

Endoscopy_UCTN_Code_TTT_1AS_2AD

## Notes

Endoscopy E-Videos https://eref.thieme.de/e-videos
*E-Videos* is an open access online section of the journal *Endoscopy* , reporting on interesting cases and new techniques in gastroenterological endoscopy. All papers include a high-quality video and are published with a Creative Commons CC-BY license. Endoscopy E-Videos qualify for HINARI discounts and waivers and eligibility is automatically checked during the submission process. We grant 100% waivers to articles whose corresponding authors are based in Group A countries and 50% waivers to those who are based in Group B countries as classified by Research4Life (see: https://www.research4life.org/access/eligibility/ ). This section has its own submission website at https://mc.manuscriptcentral.com/e-videos .
